# Reporting Melanoma: A Nationwide Surveillance of State Cancer Registries

**DOI:** 10.1155/2015/904393

**Published:** 2015-12-29

**Authors:** Kehinde O. Raji, Lauren Payne, Suephy C. Chen

**Affiliations:** ^1^Department of Internal Medicine, Scripps Clinic/Scripps Green Hospital, 10666 N. Torrey Pines Road, La Jolla, CA 92037, USA; ^2^Department of Dermatology, Howard University, 2041 Georgia Avenue NW, Suite 2107, Washington, DC 20060, USA; ^3^Division of Dermatology, Atlanta VAMC, 1670 Clairmont Road, Decatur, GA 30033, USA; ^4^Department of Dermatology, Emory University, 1525 Clifton Road NE, 3rd Floor, Atlanta, GA 30322, USA

## Abstract

The goal of our study was to determine current melanoma reporting methods available to dermatologists and dermatopathologists and quantify changes in reporting methods from 2012 to 2014. A cross-sectional study design was utilized consisting of website perusal of reporting procedures, followed up by telephone and email inquiry of reporting methods from every state cancer registry. This study was conducted over a six-month period from February to August 2014. A previous similar survey was conducted in 2012 over the same time frame and results were compared. Kansas state cancer registry provided no data. As of August 2014, 96% of 49 state cancer registries had electronic methods available to all designated reporters. Seven (14%) states required an electronic-only method of reporting melanoma cases. Eighty-six percent allowed hard copy pathology report submission. Compared to the 2012 survey, 2 additional states were found to have initiated electronic reporting methods by 2014. In conclusion, a variety of methods exist for reporting diagnosed melanoma cases. Although most state cancer registries were equipped for electronic transmission of cases for mandated reporters, a number of states were ill-equipped for electronic submission from outpatient dermatologists. There was a general trend towards electronic versus nonelectronic reporting from 2012 to 2014.

## 1. Introduction

Over the last 10 years, incidence rates of melanoma have increased for both men and women in all age groups, and, according to the United States Cancer Statistics report, there will be an estimated 65,647 melanoma cases in 2014 [1, 2]. Previous studies attributed the increased rates to increased awareness and detection of thin (≤1 mm), perhaps less aggressive, lesions [3–5]. However, more recent studies have found a statistically significant increase in incidence for tumors of all varying histological subtypes and thickness, including tumors >4 mm, indicating that the incidence rates are a true increase [6, 7]. Melanoma, along with other cancers (not including basal or squamous cell carcinomas), is mandated by law to be reported by all physicians and treatment facilities to state cancer registries. However, reporting has been particularly challenging. The advent of new techniques such as Mohs micrographic surgery and the high percentage of melanomas found to be thin lesions, approximately 70% of invasive melanomas reported to the SEER in last decade, have led to decentralization of melanoma management from hospitals to outpatient settings [8, 9]. Also, given the relative ease of access to the skin, the cycle of care may not involve a hospital, as many dermatologists now utilize outpatient-based dermatopathologists and surgical options [10]. Most of the data compiled by state cancer registries are obtained from hospital cancer registries. Therefore, a lack of or delay in detection of many thin melanomas is likely to occur, resulting in underreporting of early stage melanoma [2, 8, 11]. Generating inaccurate trend estimates on melanoma incidence and burden will undermine efforts to effectively allocate resources and implement public health initiatives [8]. It is therefore crucial to focus resources on improving surveillance and reporting methods.

Awareness of melanoma underreporting is emerging. A survey at a national conference found that 50% of dermatologists were unaware of mandated melanoma reporting and about 56% do not report newly diagnosed cases [12]. Tools to assist the practitioner awareness of methods to report to their state cancer registry may help narrow the knowledge and practice gaps. The purpose of this study is to generate a cross-sectional snapshot of the various reporting methods available to hospitals, outpatient dermatologists, and outpatient pathology labs for reporting diagnosed melanoma cases to their individual state cancer registries. Also, to investigate the dynamics of available reporting methods, we compared this snapshot to data that we obtained in 2012.

## 2. Methods

As this was publicly available information, IRB approval was not required. In order to accurately capture various reporting methods available to hospitals, independent pathology laboratories, and nonhospital affiliated physicians, we utilized a two-pronged approach. Initially, 50 state cancer registry websites were perused for reporting procedures. After this initial investigation, a brief query was made to every state cancer registry by either email or telephone inquiring about (1) available electronic reporting methods, (2) downloadable online reporting forms, and (3) submission of hard copy pathology reports. The contact information for each state cancer registry was obtained from their individual websites. “Hospital” here encompasses hospital-based pathology labs and hospital-affiliated physicians. “Pathology labs” and “physicians” refer to independent, non-hospital-affiliated entities. From the information gathered, a table of our data was generated depicting various reporting methods available to hospitals, independent pathology labs, and non-hospital-affiliated physicians. An identical survey that was conducted in 2012 was also perused to observe whether there had been a national shift towards electronic reporting methods for melanoma compared to our current survey.

## 3. Results

**Figure 1:**
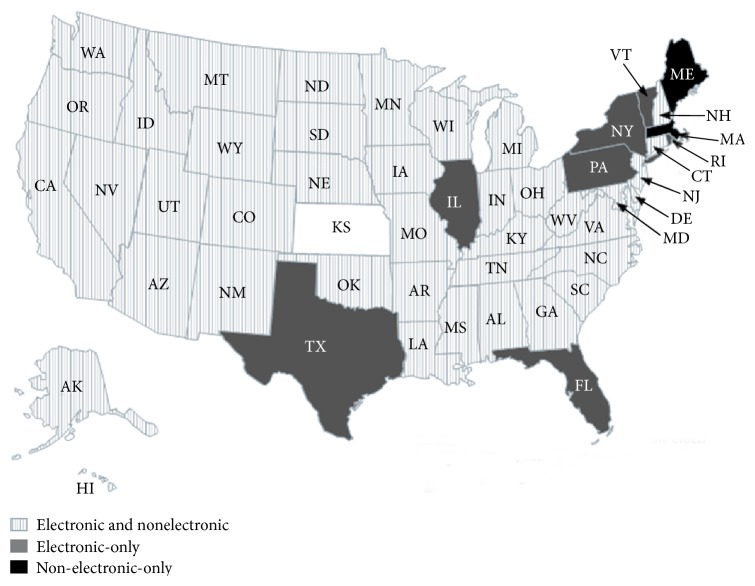
Distribution of reporting methods across state central cancer registries. Depiction of state cancer registries that allow reports of diagnosed melanoma cases in both an electronic or nonelectronic format, states that permit only electronic transmission, and states that are yet to have capabilities for electronic transmission.

All US states were successfully contacted except Kansas despite multiple attempts. Forty-seven (96%) were equipped for electronic transmission of confirmed melanoma cases from hospitals, pathology laboratories, and physicians (Table 1). Maine and Massachusetts were the only 2 states that had yet to implement an electronic method of melanoma reporting (Table 1). Seven (14%) states accepted electronic-only transmittal methods while 40 states (82%) had options for both electronic and nonelectronic reporting methods such as submission of hardcopy pathology reports (Figure 1).

For states that allowed nonelectronic reporting, the alternative methods available to mandated reporters included submission of hard copy pathology reports, registries actively requesting pathology reports of diagnosed cases, and allowing reporters to request melanoma reporting forms. Forty-two (86%) state cancer registries permitted submission of hard copy pathology reports (Table 1). Twenty-seven (55%) state cancer registries actively requested pathology reports from dermatology practices by sending out periodic surveys (Table 1). Nine (18%) states allowed dermatologists to request melanoma reporting forms directly from them (Table 1), and 22 (45%) states had reporting forms available for download on their registry websites (Table 1).

Comparison data exploring shifts in allowable reporting methods showed an increase in electronic reporting methods. In 2012, 4 state cancer registries had non-electronic-only methods for reporting melanoma. In comparison, only 2 states were noted to have such restrictions during 2014. Also, since 2012, three additional state cancer registries have joined four others in requiring an electronic-only reporting method.

## 4. Discussion

The implementation of the National Program of Cancer Registries (NPCR) in 1992 signified a push to expand and augment state cancer registries and was a crucial step in developing a system that permitted up-to-date cancer surveillance. This, in turn, enhanced research efforts to shed light on cancer distribution, population heterogeneity, and environmental etiologies, all required for more efficient resource allocation for education and screening purposes [13–15]. To ensure that these registries obtain accurate and comprehensive data, it is crucial to recognize gaps in data reporting and generate tools targeted at minimizing this gap as much as possible. To address clinician knowledge and practice gaps, an easy-to-reference table depicting reporting method options may facilitate better data acquisition and thus more robust estimates of melanoma incidence. We propose that these data be made available on the American Academy of Dermatology (AAD) website.

Given the mandate of electronic health records, it is natural to expect that all state registries eventually adopt electronic methods for melanoma reporting. There was a variation in the actual methods each state registry utilized for data transmissions. Some state registries had developed their own software, while, more interestingly, some had implemented the ability for providers eligible for Meaningful Use stage 2 (MU-2) to report melanoma by transmitting their electronic health records directly to the registries. This latter method sounds the most feasible and efficient given the ever-increasing administrative burden on physician practices. However, such a streamlined process faces several challenges given that several facets have yet to be implemented including promotion of physician reporting, on-boarding new providers, and declaration of readiness from state cancer registries. The comparison data from 2012 and 2014 demonstrated an increased shift towards electronic reporting methods by more state registries; however this shift is mostly reflective of hospitals and independent pathology labs being able to report electronically. Outpatient dermatologists still do not have the means to report melanoma cases electronically in 11 states (data not shown) but we speculate a trend towards mandated electronic reporting from clinicians.

The main limitation of our study is the cross-sectional nature of data collection. While the purpose was to create a tool depicting state-specific reporting for dermatologists and independent pathology labs, to be most effective, there should be an ongoing, real-time update such that consumers (dermatologists and pathology labs) can reference current information. Until such a tool can be developed, depicting our data on the publicly accessible website of a national organization such as AAD will hopefully lead to ease of reporting. At the minimum, our results can be utilized in educational efforts of clinicians and dermatopathologists. These efforts will hopefully lead to increased melanoma reporting.

## Figures and Tables

**Table 1:** Reporting methods available to mandated melanoma reporters across US state cancer registries.

States (all except Kansas)	Electronic reporting method available	Electronic-only	Non-electronic-only	Hardcopy path reports	Registry requests reports	Provider request reporting forms	Downloadable forms online
Alabama	‡			‡	*∗*	‡	*∗*
Alaska	‡			‡	*∗*	*∗*	‡
Arizona	‡			‡	‡	*∗*	‡
Arkansas	‡			‡	‡	*∗*	‡
California	‡			‡	‡	*∗*	‡
Colorado	‡			‡	‡	‡	*∗*
Connecticut	‡			‡	‡	*∗*	*∗*
Delaware	‡			‡	*∗*	*∗*	‡
Florida	‡	‡		*∗*	*∗*	*∗*	*∗*
Georgia	‡			‡	*∗*	*∗*	*∗*
Hawaii	‡			‡	‡	*∗*	*∗*
Idaho	‡			‡	‡	*∗*	‡
Illinois	‡	‡		*∗*	*∗*	*∗*	*∗*
Indiana	‡			‡	*∗*	‡	‡
Iowa	‡			‡	‡	‡	*∗*
Kentucky	‡			‡	‡	*∗*	*∗*
Louisiana	‡			‡	‡	*∗*	*∗*
Maine	*∗*		‡	‡	*∗*	*∗*	‡
Maryland	‡			‡	‡	*∗*	‡
Massachusetts	*∗*		‡	‡	‡	‡	*∗*
Michigan	‡			‡	*∗*	*∗*	‡
Minnesota	‡			‡	‡	*∗*	*∗*
Mississippi	‡			‡	‡	‡	*∗*
Missouri	‡			‡	*∗*	*∗*	‡
Montana	‡			‡	‡	*∗*	‡
Nebraska	‡			‡	‡	‡	*∗*
Nevada	‡			‡	‡	‡	‡
New Hampshire	‡			‡	‡	*∗*	‡
New Jersey	‡			‡	*∗*	*∗*	‡
New Mexico	‡			‡	‡	*∗*	*∗*
New York	‡	‡		*∗*	*∗*	*∗*	*∗*
North Carolina	‡			‡	‡	*∗*	‡
North Dakota	‡			‡	‡	*∗*	*∗*
Ohio	‡			‡	*∗*	*∗*	‡
Oklahoma	‡			‡	*∗*	*∗*	*∗*
Oregon	‡			‡	*∗*	*∗*	‡
Pennsylvania	‡	‡		*∗*	*∗*	*∗*	*∗*
Rhode Island	‡	‡		*∗*	*∗*	*∗*	*∗*
South Carolina	‡			‡	‡	‡	*∗*
South Dakota	‡			‡	‡	*∗*	*∗*
Tennessee	‡			‡	‡	*∗*	*∗*
Texas	‡	‡		*∗*	*∗*	*∗*	*∗*
Utah	‡			‡	‡	*∗*	‡
Vermont	‡	‡		*∗*	*∗*	*∗*	*∗*
Virginia	‡			‡	*∗*	*∗*	‡
Washington	‡			‡	‡	*∗*	*∗*
West Virginia	‡			‡	*∗*	*∗*	‡
Wisconsin	‡			‡	*∗*	*∗*	‡
Wyoming	‡			‡	‡	*∗*	*∗*
Total *N* (%)	47 (96)	7 (14)	2 (0.04)	42 (86)	27 (55)	9 (18)	22 (45)

‡Available; *∗*not available.

The “Electronic reporting method available” column elucidates the state cancer registries that have the capabilities for electronic reporting of diagnosed melanoma cases. The next two columns depict those states that allow an electronic-only transmission method and states that do not yet have capabilities for electronic transmission. The last four columns depict methods utilized by state cancer registries that allowed nonelectronic reporting. These include submission of hard copy path reports, periodic surveys to request pathology reports from practitioners, allowing providers to request melanoma reporting forms directly from their state cancer registries, and having melanoma reporting forms available for download on their websites.

## Abbreviations

CDC: Center for Disease Control
NCPR: National Program of Cancer Registries
NCI: National Cancer Institute
MU-2: Meaningful Use stage 2
SEER: Surveillance, Epidemiology, and End Results.
